# Pharmacokinetics of diluted (U20) insulin aspart compared with standard (U100) in children aged 3–6 years with type 1 diabetes during closed-loop insulin delivery: a randomised clinical trial

**DOI:** 10.1007/s00125-014-3483-6

**Published:** 2014-12-24

**Authors:** Yue Ruan, Daniela Elleri, Janet M. Allen, Martin Tauschmann, Malgorzata E. Wilinska, David B. Dunger, Roman Hovorka

**Affiliations:** 1 Department of Paediatrics, University of Cambridge, Cambridge, UK; 2 University of Cambridge Metabolic Research Laboratories and National Institute for Health Research Cambridge Biomedical Research Centre, Level 4, Wellcome Trust–MRC Institute of Metabolic Science, Box 289, Addenbrooke’s Hospital, Hills Rd, Cambridge, CB2 0QQ UK

**Keywords:** Aspart, Insulin absorption, Insulin concentration, Pharmacokinetics, Rapid-acting insulin, Type 1 diabetes, Young children

## Abstract

**Aims/hypothesis:**

The aim of this study was to compare the pharmacokinetics of two different concentrations of insulin aspart (B28Asp human insulin) in children aged 3–6 years with type 1 diabetes.

**Methods:**

Young children with type 1 diabetes underwent an open-label, randomised, two-period crossover study in a clinical research facility, 2–6 weeks apart. In random order, diluted (1:5 dilution with saline [154 mmol/l NaCl]; 20 U/ml) or standard strength (100 U/ml) insulin aspart was administered via an insulin pump as a meal bolus and then overnight by closed-loop insulin delivery as determined by a model predictive algorithm. Plasma insulin was measured every 30–60 min from 17:00 hours on day 1 to 8:00 hours on day 2. We measured the time-to-peak insulin concentration (*t*_*max*_), insulin metabolic clearance rate (*MCR*_*I*_) and background insulin concentration (*ins*_*c*_) using compartmental modelling.

**Results:**

Eleven children (six male; age range 3.75–6.96 years, HbA_1c_ 7.6% ± 1.3% [60 ± 14 mmol/mol], BMI standard deviation score 1.0 ± 0.8, duration of diabetes 2.2 ± 1.0 years, total daily dose 12.9 [10.6–16.5] U, fasting C-peptide concentration 5 [5–17.1] pmol/l; mean ± SD or median [interquartile range]) participated in the study. No differences between standard and diluted insulin were observed in terms of *t*_*max*_ (59.2 ± 14.4 vs 61.6 ± 8.7) min for standard vs diluted, *p* = 0.59; *MCR*_*I*_ (1.98 × 10^−2^ ± 0.99 × 10^−2^ vs 1.89 × 10^−2^ ± 0.82 × 10^−2^ 1/kg/min, *p* = 0.47), and *ins*_*c*_ (34 [1–72] vs 23 [3–65] pmol/l, *p* = 0.66). However, *t*_*max*_ showed less intersubject variability following administration of diluted aspart (SD 14.4 vs 8.7 min, *p* = 0.047).

**Conclusions/interpretation:**

Diluting insulin aspart does not change its pharmacokinetics. However, it may result in less variable absorption and could be used in young children with type 1 diabetes undergoing closed-loop insulin delivery.

*Trial registration*: Clinicaltrials.gov NCT01557634

*Funding*: Funding was provided by the JDRF, 7th Framework Programme of the European Union, Wellcome Trust Strategic Award and the National Institute for Health Research Cambridge Biomedical Research Centre.

**Electronic supplementary material:**

The online version of this article (doi:10.1007/s00125-014-3483-6) contains peer-reviewed but unedited supplementary material, which is available to authorised users.

## Introduction

Rapid-acting insulin analogues are commonly selected for insulin pump therapies to achieve more physiological postprandial insulin levels. Further acceleration of absorption is desirable to reduce postprandial glucose excursions, which are often followed by late postprandial hypoglycaemia, and to improve the performance of closed-loop insulin delivery systems [1]. This may be particularly relevant in younger children, where postprandial swings in glucose levels may be greater and the consequences of hypoglycaemia may be severe.

Diluting regular human insulin may accelerate its absorption [2, 3] although the exact mechanism responsible for this is unknown. The formation of hexamers may be reduced, thus promoting absorption from the subcutaneous depot to the surrounding capillary bed. Rapid-acting insulin analogues prevent the formation of hexamers and generally provide more favourable pharmacokinetics [3]. However, little is known about whether dilution affects insulin analogue absorption. An exploratory study in pigs showed no difference in the absorption of insulin aspart (B28Asp human insulin) at three different concentrations delivered via an insulin pump [4], but no human studies have been reported. The objective of the present study was to evaluate aspart pharmacokinetics in young children following the administration of standard strength (100 U/ml) and diluted (20 U/ml) insulin.

## Methods

### Participants

**Fig. 1:**
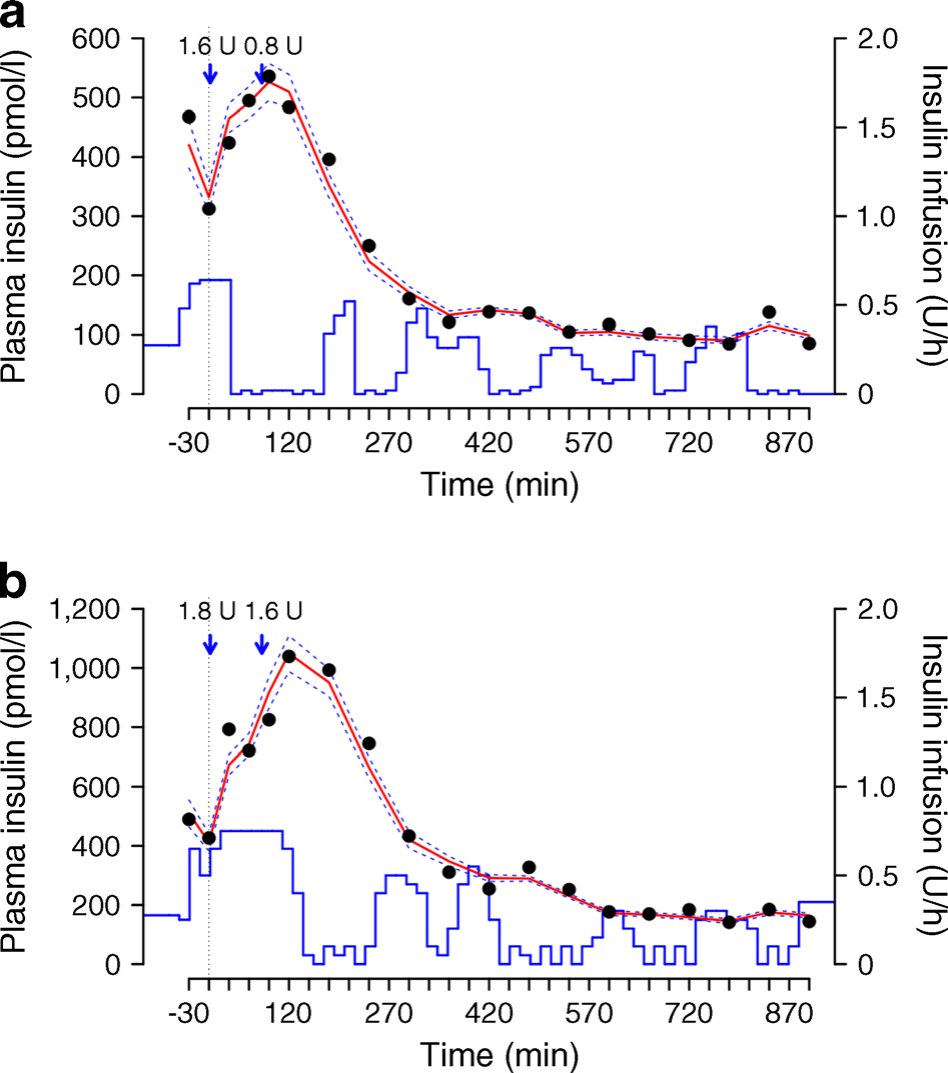
Model fit to plasma insulin concentration in a sample participant during the delivery of diluted (**a**) and standard strength (**b**) insulin. Evening meals were consumed at time 0 and were accompanied by split prandial insulin boluses, indicated by blue arrows. The dotted vertical line indicates time that closed-loop delivery started. The red solid line shows the median of the fitted model. The blue dashed lines show the 95% credible interval of the fitted model. The solid blue line shows insulin infusion

We analysed data from 11 pump-treated children with type 1 diabetes (six male, age 5.07 ± 1.12 years [range 3.75–6.96 years], HbA_1c_ 7.6% ± 1.3% (60 ± 14 mmol/mol), BMI standard deviation score 1.0 ± 0.8 [range −0.55 to 2.11], duration of diabetes 2.2 ± 1.0 years [range 0.7–3.7 years], total daily dose 12.9 [10.6–16.5] U/day and 0.65 [0.59–0.69] U/kg/day; median [interquartile range (IQR)]) participating in a study evaluating closed-loop insulin delivery [5]. Electronic supplementary material (ESM) Fig. 1 shows the flow of participants through the study. All participants agreed to take part in the study and their parent or carer provided informed consent prior to the commencement of clinical trials. The study was approved by the Cambridge Central Research Ethics Committee.

### Study protocol

Children were admitted in the afternoon on two study visits, 2–6 weeks apart, at the Wellcome Trust Clinical Research Facility, Addenbrooke’s Hospital, Cambridge, to undergo closed-loop insulin delivery from 17:00 hours until 8:00 hours the following morning. On both occasions, the children consumed identical evening meals at 17:00 hours [44 ± 12 g carbohydrate] and optional bedtime snacks [6 ± 7 g carbohydrate]. Standard pump bolus calculators were used to calculate meal insulin boluses and basal rates on the insulin pump (Animas 2020, Johnson & Johnson, PA, USA) were adjusted every 15 min according to the output of a model predictive control algorithm [6] informed by real-time continuous glucose monitoring values (Dexcom G4, Dexcom, CA, USA). Standard strength (100 U/ml) insulin aspart (Novo Nordisk, Bagsvaerd, Denmark) was infused at one visit and diluted aspart (0.9% saline [154 mmol/l NaCl] at 1:5 ratio, 20 U/ml) was administered at the other; the order of the two interventions was random. During closed-loop administration of diluted insulin, the pump settings were changed by multiplying the current settings by a factor of five, and the revised settings were entered into the computer running the closed-loop algorithm.

### Insulin and C-peptide measurement

Venous blood samples were taken at −30, 0, 30, 60, 90, 120, 180, 240, 300, 360, 420, 480, 540, 600, 660, 720, 780, 840 and 900 min relative to 17:00 hours for measuring the plasma insulin concentration. Plasma was separated by centrifugation immediately after sampling. Plasma insulin was measured using an immunochemiluminometric assay (Invitron, Monmouth, UK; intra-assay CV 4.7%, interassay CV 7.2–8.1%; 100% cross reactivity with insulin aspart). Fasting C-peptide levels were measured at plasma glucose values above 6 mmol/l by ELISA (Mercodia, Uppsala, Sweden).

### Pharmacokinetics

Aspart pharmacokinetics was assessed using a two-compartment model [7, 8] measuring the time-to-peak of plasma insulin concentration (*t*_*max*_), metabolic clearance rate of insulin (*MCR*_*I*_), and background plasma insulin concentration (*ins*_*c*_). The three parameters were estimated by fitting a Bayesian statistical model to plasma insulin data (ESM Methods). The compartmental model was implemented using WinBUGS software version 1.4 (MRC Biostatistics Unit, Cambridge, UK) [9] with a WBDev interface (MRC Biostatistics Unit). The insulin measurement error was assumed to be uncorrelated and normally distributed, with a CV of 6%.

### Statistical analysis

Differences between pharmacokinetic parameters were assessed by paired *t* test (for normally distributed variables) or the Wilcoxon signed-rank test (for non-normally distributed variables). Levene’s test was used to assess the equality of variances of parameter estimates. The Spearman’s rank correlation evaluated correlations between pharmacokinetic and demographic indices. A *p* value of <0.05 was considered statistically significant. Values are presented as the mean ± SD or the median (IQR) unless stated otherwise.

## Results

Figure 1 demonstrates a model fitted to the plasma insulin concentration in a sample participant (ESM Fig. 2). Plasma insulin increased rapidly after a prandial insulin bolus and then decreased to basal levels and was variable overnight, reflecting insulin delivery informed by the control algorithm. Weighted residuals demonstrated an overall good and unbiased fit across all participants (ESM Fig. 3).

Table 1 shows the estimates of three pharmacokinetic parameters (*t*_*max*_, *MCR*_*I*_ and *ins*_*c*_) measured after standard strength and diluted insulin aspart administration. No differences between the two preparations were observed. However, a smaller intersubject variability in *t*_*max*_ was observed following the administration of diluted insulin (SD 14.4 vs 8.7 min, standard vs diluted; *p* = 0.047).

**Table 1:** Comparison of parameter estimates between diluted and standard strength insulin delivery

Model parameters	Standard strength insulin 100 U/ml (*N* = 11)	Diluted insulin 20 U/ml (*N* = 11)	*p* value
*t*_*max*_ (min)	59.2 ± 14.4	61.6 ± 8.7	0.59^a^
*MCR*_*I*_ (10^−2^ × 1/kg/min)	1.98 ± 0.99	1.89 ± 0.82	0.47^a^
*ins*_*c*_ (pmol/l)	34 (1–72)	23 (3–65)	0.66^b^

^a^Paired *t* test; data are means ± SD

^b^Wilcoxon signed rank test; data are medians (IQR)

Relationships between the pharmacokinetic parameters and demographic variables are shown in ESM Table 1. The *ins*_*c*_ parameter, but not the others, correlated positively with the insulin total daily dose (*r*_*s*_ = 0.71, *p* = 0.014), but not with the fasting C-peptide concentration (*r*_*s*_ = −0.19, *p* = 0.39; C-peptide concentration 5 [3.7–17.1] pmol/l, median [IQR]), suggesting that the *ins*_*c*_ does not reflect residual insulin secretion.

## Discussion

Our data from a randomised, two-period crossover study comparing closed-loop insulin delivery using diluted vs standard insulin aspart in young children with type 1 diabetes suggest no significant differences in aspart pharmacokinetics between the two strengths except for reduced intersubject variability in *t*_*max*_ following administration of diluted aspart.

The mean value of *t*_*max*_ in the present study (60.4 min) is comparable with that reported in older children and adults with type 1 diabetes following insulin bolus injection (49 min) [10] or during closed-loop insulin delivery (66 min and 51 min, respectively) [7, 8]. Similar estimates of *MCR*_*I*_ and *ins*_*c*_ were also found in the current study and published literature (1.94 vs 1.68 × 10^−2^ vs 1.90 × 10^−2^ 1/kg/min for *MCR*_*I*_, 29 vs 28 vs 61 pmol/l for *ins*_*c*_) [7, 8]. In the present study, *MCR*_*I*_ values in the two visits were highly correlated (*r* = 0.918, *p* < 0.001), as were the *ins*_*c*_ values (*r*_*s*_ = 0.964, *p* < 0.001), indicating that these characteristics are reproducible even with diluted insulin. However, this association was not found for *t*_*max*_ (*p* = 0.4).

Our main finding that no difference in aspart pharmacokinetics exists between standard-strength and diluted insulin administered to young children is consistent with Petersen et al, who demonstrated no significant difference between U200, U100 and U20 in terms of insulin aspart pharmacokinetics during continuous subcutaneous insulin infusion in pigs [4]. Theoretical calculations considering hexamer, dimer and monomer formation and absorption characteristics also suggested no effect of dilution on the absorption of rapid-acting insulin analogues [11]; in contrast, diluted regular insulin is absorbed more rapidly in humans [3].

We show less variable absorption of diluted insulin, based on a difference in the intersubject variability of *t*_*max*_. The origin of such reduced variability is unclear. We hypothesise that it may be related to fewer mechanical pump delivery errors and more consistent absorption from the insulin depot because of its larger size. The lower absorption variability might benefit young children by allowing caregivers to more confidently predict the timing of onset and the duration of insulin action, and providing more consistent glycaemic results during closed-loop insulin delivery.

Our data suggest that background *ins*_*c*_ does not reflect residual insulin secretion, as hypothesised previously [7]. In the present study, the fasting C-peptide concentration was lower than the detection limit of 5 pmol/l in more than half of the participants, and did not correlate with *ins*_*c*_. However, the significant positive correlation between *ins*_*c*_ and the total daily insulin dose suggests that the background insulin level may result from a slowly absorbing insulin pool in the subcutaneous insulin depot, where the amount of ‘residual’ insulin is proportional to one’s total daily dose. However, this hypothesis is derived from our retrospective analysis on data from a limited sample size and further investigations are warranted.

The strength of our study is the evaluation of insulin kinetics in an underserved population of young children with type 1 diabetes who may benefit from insulin dilution through reduced variability in insulin absorption. The limitations are the relatively small size of the study and the use of saline instead of a proprietary diluter, which is not generally available. The sampling frequency of 30–60 min is lower than that used by others [2], but this is compensated for by longer visits and the dynamic nature of insulin delivery which enables accurate parameter estimates to be obtained, as demonstrated by computer simulations (ESM Simulation analysis).

In conclusion, we found no significant change in pharmacokinetics when using diluted aspart. However, diluting aspart may lead to less variable absorption and could be beneficial to young children with type 1 diabetes undergoing closed-loop insulin delivery.

## Electronic supplementary material

Below is the link to the electronic supplementary material.

ESM Methods (PDF 26 kb)

ESM Fig. 1 (PDF 31 kb)

ESM Fig. 2 (PDF 2286 kb)

ESM Fig. 3 (PDF 137 kb)

ESM Simulation analysis (PDF 187 kb)

ESM Table 1 (PDF 12 kb)

## Abbreviations

*ins*_*c*_: Background insulin concentration
IQR: Interquartile range
*MCR*_*I*_: Insulin metabolic clearance rate
*t*_*max*_: Time-to-peak insulin concentration
