# Supplementary material for: Pharmacokinetics of diluted (U20) insulin aspart compared with standard (U100) in children aged 3–6 years with type 1 diabetes during closed-loop insulin delivery: a randomised clinical trial
Source: Diabetologia. 2014 Dec 24;58(4):687–90. doi: 10.1007/s00125-014-3483-6 (PMC4351431; doi:10.1007/s00125-014-3483-6)
Supplement: Supplementary file 1 — (PDF 26 kb) [file 125_2014_3483_MOESM1_ESM.pdf]

ESM Methods

The pharmacokinetic parameters were estimated using compartment modelling. In the present study, a two-compartment model described insulin aspart kinetics as in our previous work [1-4]:

$$\frac{di_1(t)}{dt} = u(t) - \frac{i_1(t)}{t_{\max}} \qquad (1)$$

$$\frac{di_2(t)}{dt} = -\frac{1}{t_{\max}}\left(i_2(t) - i_1(t)\right) \qquad (2)$$

where $i_1(t)$ and $i_2(t)$ is the amount of insulin in the two subcutaneous insulin depots (U) and $u(t)$ denotes the exogenous insulin delivery (U/h; model input) representing jointly prandial insulin bolus (appropriately represented as a short high-burst insulin infusion) or basal insulin infusion entering the compartment i_1 and then the insulin is transferred into the compartment i_2 at a fractional rate of $1/t_{max}$, where t_{max} is the time-to-peak of plasma insulin (min).

The plasma insulin concentration is obtained assuming instantaneous equilibration between insulin appearance in plasma and plasma insulin reflecting a short plasma insulin half-life relative to the frequency of plasma insulin measurements:

$$I_{plasma}(t) = \frac{1000}{t_{\max} MCR_I W} i_2(t) + ins_c \qquad (3)$$

where $I_{plasma}(t)$ is the plasma insulin concentration (mU/l; model output), MCR_I is the metabolic clearance rate of insulin (l/kg/min), ins_c is the background insulin (mU/l) which is assumed constant throughout the study period and may explain insulin concentration due to previously unabsorbed insulin left in the insulin depot, and W is subject's weight (kg).

t_{max} , MCR_I and ins_c are the individual pharmacokinetic (PK) model parameters estimated by fitting a Bayesian statistical model to each subject's data set.

To introduce the Bayesian approach, let $\mathbf{p_i} = [t_{max,i}\ MCR_{I,i}\ ins_{c,i}]$ represent the PK parameter vector for subject i and $\boldsymbol{y_i}$ the plasma insulin concentration data set for subject i. According to the Bayes theory,

$$f(\mathbf{p_i} \mid \mathbf{y_i}) = \frac{f(\mathbf{y_i} \mid \mathbf{p_i})\, f(\mathbf{p_i})}{f(\mathbf{y_i})} \propto f(\mathbf{y_i} \mid \mathbf{p_i})\, f(\mathbf{p_i}) \qquad (4)$$

where $f(\mathbf{p_i} \mid \mathbf{y_i})$ is the posterior density of the unknown parameter $\mathbf{p_i}$, $f(\mathbf{y_i} \mid \mathbf{p_i})$ is the likelihood function, $f(\mathbf{p_i})$ is the prior density of $\mathbf{p_i}$, and $f(\mathbf{y_i})$ performs as a scale factor. $f(\mathbf{y_i} \mid \mathbf{p_i})$ is determined by solving Eq. (1) and (2) and calculating the plasma insulin concentration using Eq. (3). The model was numerically identified using WinBUGS (Bayesian inference Using Gibbs Sampling for Windows) software version 1.4 (MRC Biostatistics Unit, Cambridge, UK). We derived a closed-form solution of the model given in Eqs. (1)-(3). The WinBUGS extension 'WBDev' was utilized to implement this solution into WinBUGS through a separate module.

Parameter mean/median and credible intervals can be derived from the posterior density. Median values of parameters obtained from the posterior density functions were subjected to the statistical analysis.

The prior density reflects our previous knowledge about unknown parameters. In the present study, we used vague log-normal prior distributions for PK parameters (see below) with t_{max}, MCR_I and ins_c centred at 60 min, 0.01 l/kg/min and 36 pmol/l, respectively, with a large coefficient of variation of 10^4.

Table. Uninformative prior distributions of pharmacokinetic parameters used in the Bayesian analysis.

Model parameter	Prior distribution[a]
t_{max} (min)	$\log(t_{\max}) \sim N(\log(60), 10^2)$
MCR_I (10^{-2} × l/kg/min)	$\log(MCR_I) \sim N(\log(0.01), 10^2)$
ins_c (pmol/l)	$\log(ins_c) \sim N(\log(36), 10^2)$

[a] Log-normal distributions to guarantee parameter positivity

References

[1] Goudie RJ, Lunn D, Hovorka R, Murphy HR (2014) Pharmacokinetics of insulin aspart in pregnant women with type 1 diabetes: every day is different. Diabetes Care 37: e121-122

[2] Haidar A, Elleri D, Kumareswaran K, et al. (2013) Pharmacokinetics of insulin aspart in pump-treated subjects with type 1 diabetes: reproducibility and effect of age, weight, and duration of diabetes. Diabetes Care 36: e173-174

[3] Haidar A, Duval C, Legault L, Rabasa-Lhoret R (2013) Pharmacokinetics of Insulin Aspart and Glucagon in Type 1 Diabetes during Closed-Loop Operation. J Diabetes Sci Technol 7: 1507-1512

[4] Ruan Y, Thabit H, Kumareswaran K, Hovorka R (2014) Pharmacokinetics of insulin lispro in type 2 diabetes during closed-loop insulin delivery. Comput Methods Programs Biomed 117: 298-307
