# Supplementary material for: Pharmacokinetics of diluted (U20) insulin aspart compared with standard (U100) in children aged 3–6 years with type 1 diabetes during closed-loop insulin delivery: a randomised clinical trial
Source: Diabetologia. 2014 Dec 24;58(4):687–90. doi: 10.1007/s00125-014-3483-6 (PMC4351431; doi:10.1007/s00125-014-3483-6)

ESM Fig. 1. Flow of participants (CONSORT Flow Diagram) through the study comparing aspart pharmacokinetics using diluted insulin and standard insulin.

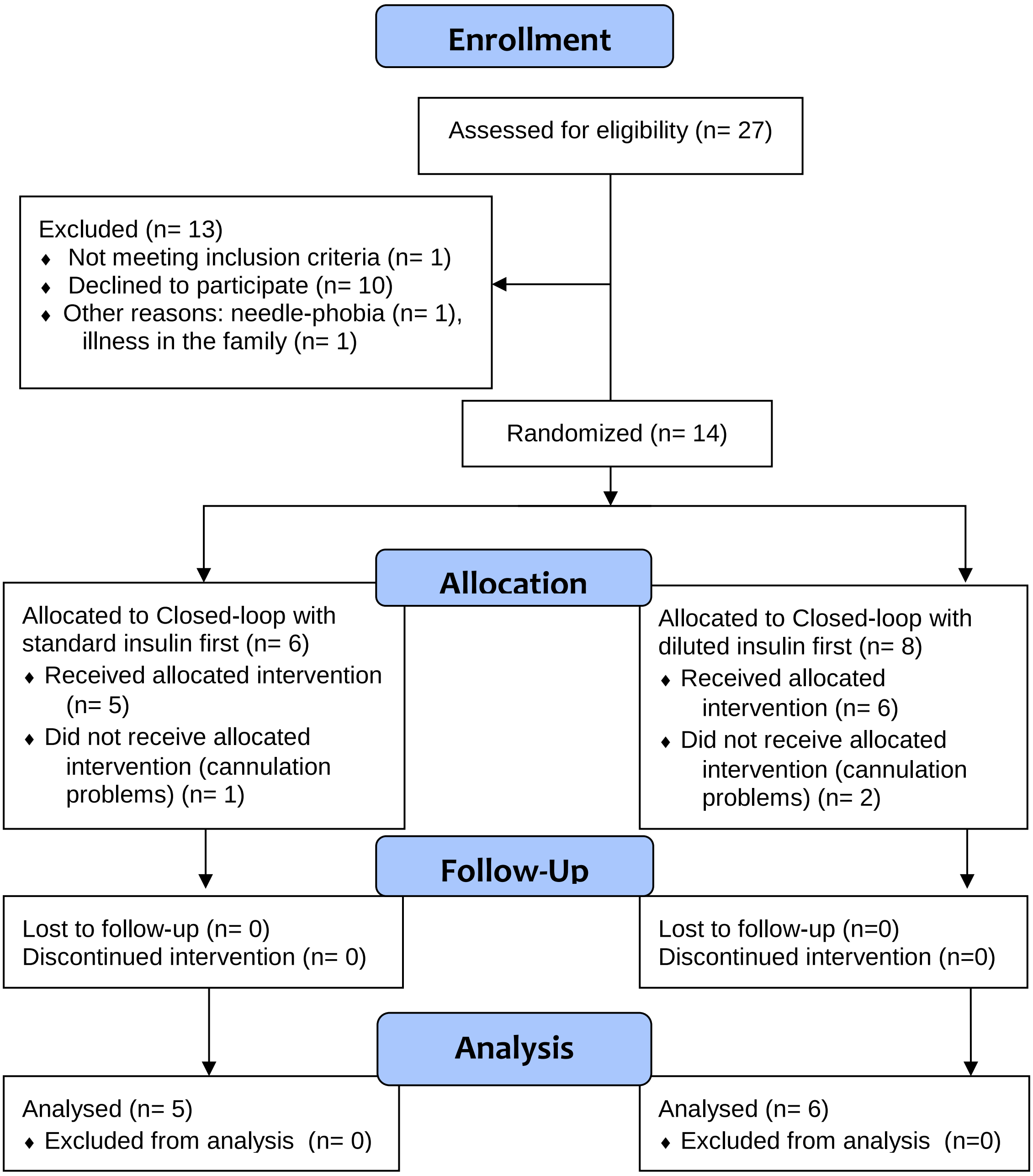

Supplement: Supplementary file 2 — (PDF 31 kb) [file 125_2014_3483_MOESM2_ESM.pdf]
