# Supplementary material for: Pharmacokinetics of diluted (U20) insulin aspart compared with standard (U100) in children aged 3–6 years with type 1 diabetes during closed-loop insulin delivery: a randomised clinical trial
Source: Diabetologia. 2014 Dec 24;58(4):687–90. doi: 10.1007/s00125-014-3483-6 (PMC4351431; doi:10.1007/s00125-014-3483-6)
Supplement: Supplementary file 3 — (PDF 2286 kb) [file 125_2014_3483_MOESM3_ESM.pdf]

ESM Fig. 2. Model fit to plasma insulin concentration during the delivery of diluted (a) and standard strength (b) insulin aspart. Evening meals were consumed at time 0 and were accompanied by prandial insulin boluses indicated by blue down arrow(s). The dotted vertical line indicates time when closed-loop started. The red solid line shows median of model fit. The blue dashed lines show 95% credible interval of model fit. The blue solid line shows insulin infusion. Pharmacokinetic parameter estimates are given at the top of the plots.

a

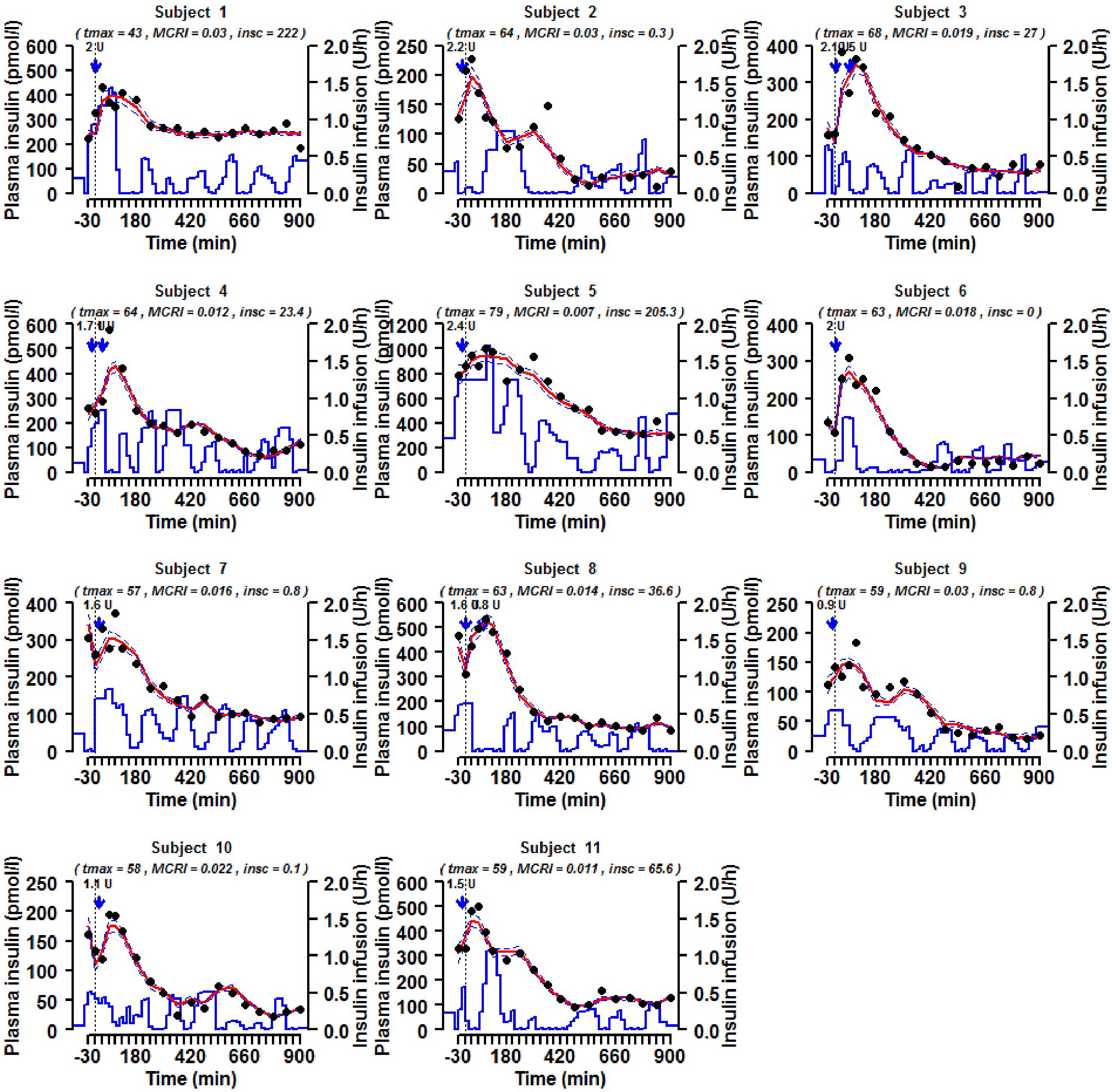

b

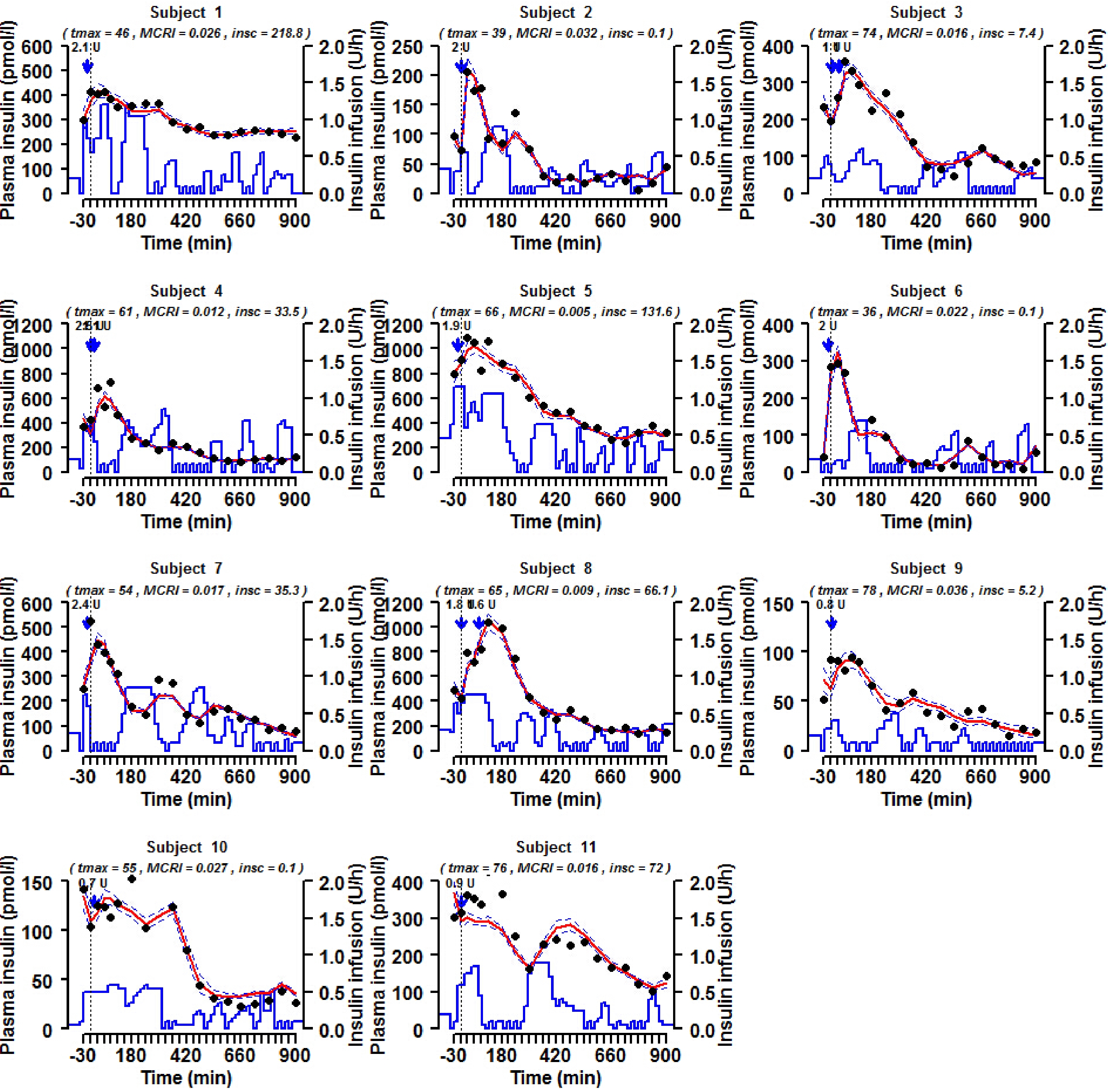
Subject 1
(tmax = 46 , MCRI = 0.026 , insc = 218.8)
2.1 U
Subject 2
(tmax = 39 , MCRI = 0.032 , insc = 0.1)
2 U
Subject 3
(tmax = 74 , MCRI = 0.016 , insc = 7.4)
Subject 4
(tmax = 61 , MCRI = 0.012 , insc = 33.5)
Subject 5
(tmax = 66 , MCRI = 0.005 , insc = 131.6)
1.9 U
Subject 6
(tmax = 36 , MCRI = 0.022 , insc = 0.1)
2 U
Subject 7
(tmax = 54 , MCRI = 0.017 , insc = 35.3)
2.4 U
Subject 8
(tmax = 65 , MCRI = 0.009 , insc = 66.1)
1.8 U
0.6 U
Subject 9
(tmax = 78 , MCRI = 0.036 , insc = 5.2)
0.8 U
Subject 10
(tmax = 55 , MCRI = 0.027 , insc = 0.1)
0.7 U
Subject 11
(tmax = 76 , MCRI = 0.016 , insc = 72)
0.9 U
Plasma insulin (pmol/l)
Insulin infusion (U/h)
Time (min)
