# Supplementary material for: Pharmacokinetics of diluted (U20) insulin aspart compared with standard (U100) in children aged 3–6 years with type 1 diabetes during closed-loop insulin delivery: a randomised clinical trial
Source: Diabetologia. 2014 Dec 24;58(4):687–90. doi: 10.1007/s00125-014-3483-6 (PMC4351431; doi:10.1007/s00125-014-3483-6)

ESM Fig. 3. Weighted residuals (difference between model and measured plasma insulin concentration divided by the measurement error) with diluted insulin (top panel) and standard insulin strength (bottom panel) (median [IQR]; N=11).

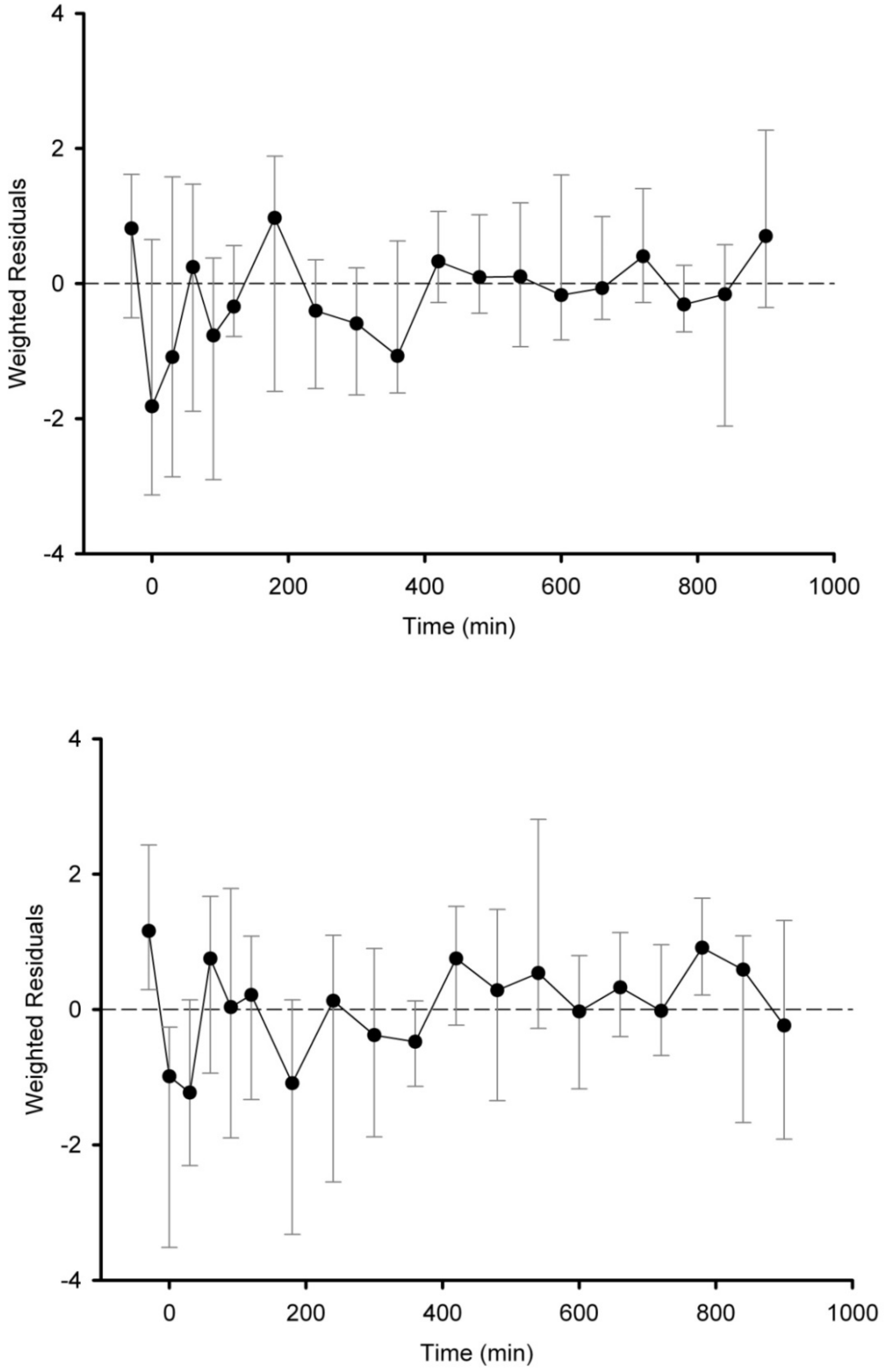

Supplement: Supplementary file 4 — (PDF 137 kb) [file 125_2014_3483_MOESM4_ESM.pdf]
