# Supplementary material for: Pharmacokinetics of diluted (U20) insulin aspart compared with standard (U100) in children aged 3–6 years with type 1 diabetes during closed-loop insulin delivery: a randomised clinical trial
Source: Diabetologia. 2014 Dec 24;58(4):687–90. doi: 10.1007/s00125-014-3483-6 (PMC4351431; doi:10.1007/s00125-014-3483-6)
Supplement: Supplementary file 5 — (PDF 187 kb) [file 125_2014_3483_MOESM5_ESM.pdf]

ESM Simulation analysis

We applied the compartment analysis unlike other studies, adopting a more frequent sampling, which applied the non-compartment analysis. The compartment analysis is the method of choice when less-frequent sampling such as ours is used as all measurements contribute to the estimation of model parameters. Given the dynamic nature of the insulin delivery (so called “a variable input” experiment design) and a 15 hour experimental period compared to the standard bolus approach (so called “a unit response” experiment design) with a shorter duration, we hypothesise that the compartment analysis was able to determine with accuracy model parameters.

To support the above stated hypothesis, we carried out additional analyses using synthetic data sets to answer the two following questions:

1) Will a 15min sampling frequency improve the accuracy of parameter estimates

2) Is a 30-60min sampling frequency sufficient to detect a 10min reduction in t_{max} (reduction by 10min was chosen as it was considered clinically meaningful)

The details of the synthetic data analyses are as follows. Using (1) the compartment model described in the paper, (2) individual insulin delivery as recorded in 11 subjects during the standard strength insulin visits, and (3) parameter values from 11 subjects during the standard strength insulin visit, we created 11 synthetic error-free plasma insulin profiles. We sampled these error free time-concentration profiles every 15 min and added a measurement error as an uncorrelated zero-mean normally distributed noise with 6% CV. This created “15min sampling profiles” (11 profiles, each with 63 plasma insulin measurements). We downsampled these profiles with a 30-60min sampling frequency identical to that used in the paper. This created the “30-60min sampling profiles” (each with 19 plasma insulin measurements).

Both sets of profiles were then subjected to the parameter estimation procedure as described in the paper. The resulting parameter estimates were then compared to the “gold standard” parameter values used to generate the profiles (this answered questions 1).

In a similar fashion, we generated another “15min sampling profiles” (N = 11) and “30-60min sampling profiles” (N = 11) using t_{max} reduced by 10min. This was followed by estimating model parameters from these profiles (this answered question 2).

The results of the synthetic analyses confirm our hypothesis. Table D1 shows comparable parameter estimates between the “gold standard” and the two sampling frequencies.

Table D2 shows that the “30-60min” sampling frequency can accurately determine a difference of 10min in t_{max}. Similar results were obtained using “15min” sampling frequency as shown in Table D3. There appears no additional advantage of 15min sampling compared to the original sampling adopted in the paper.

Table D1. Comparison of parameter estimates obtained from synthetic data sets using the study sampling frequency ("30-60min sampling profiles") and 15 min sampling frequency ("15min sampling profiles"). The "gold standard" column indicates parameter values used to generate the synthetic data sets.

	Original parameter values ("gold standard") N = 11	Parameter estimates using "30-60min sampling profiles" (19 insulin measurements) N = 11	Parameter estimates using "15min sampling profiles" (63 insulin measurements) N = 11	*p* value
t_{max} (min)	59.2 (14.4)	59.5 (13.9)	60.0 (15.9)	0.89[a]
MCR_I (10^{-2} × l/kg/min)	1.98 (0.99)	1.98 (0.97)	1.97 (0.96)	0.67[a]
ins_c (mU/l)	5.6 (0.2, 12.0)	4.1 (0.1,13.5)	5.6 (0.1,12.5)	0.11[b]

[a] Two-way ANOVA
[b] Two-way ANOVA after rank normal transformation

Table D2. Parameter estimates using original t_{max} and t_{max} reduced by 10 min using synthetic data with the original "30 – 60min" sampling frequency.

	Parameter estimates using synthetic data with original[c] t_{max} N = 11	Parameter estimates using synthetic data with t_{max} minus 10 minutes N = 11	*p* value
t_{max} (min)	59.5 (13.9)	49.4 (14.6)	<0.001[a]
MCR_I (10^{-2} × l/kg/min)	1.98 (0.97)	1.99 (0.98)	0.92[a]
ins_c (mU/l)	4.1 (0.1,13.5)	4.5 (0.1,12.1)	0.53[b]

[a] Paired t-test
[b] Wilcoxon signed rank test
[c] Third column from Table D1

Table D3. Parameter estimates using original t_{max} and t_{max} reduced by 10 min using synthetic data with the "15min" sampling frequency.

	**Parameter estimates using synthetic data with original[c] t_{max} N = 11**	**Parameter estimates using synthetic data with t_{max} minus 10 minutes N = 11**	***p* value**
t_{max} (min)	60.0 (15.9)	49.8 (15.2)	<0.001[a]
MCR_I (10^{-2} × l/kg/min)	1.97 (0.96)	1.97 (0.96)	0.98[a]
ins_c (mU/l)	5.6 (0.1,12.5)	5.2 (0.1,13.2)	0.33[b]

[a] Paired t-test
[b] Wilcoxon signed rank test
[c] Fourth column from Table D1
