# Supplementary material for: Pharmacokinetics of diluted (U20) insulin aspart compared with standard (U100) in children aged 3–6 years with type 1 diabetes during closed-loop insulin delivery: a randomised clinical trial
Source: Diabetologia. 2014 Dec 24;58(4):687–90. doi: 10.1007/s00125-014-3483-6 (PMC4351431; doi:10.1007/s00125-014-3483-6)
Supplement: Supplementary file 6 — (PDF 12 kb) [file 125_2014_3483_MOESM6_ESM.pdf]

**ESM Table 1. Spearman's rank correlation coefficients between pharmacokinetic parameters and demographic characteristics (N=11).**

| | Age (yrs) | BMI ($kg/m^2$) | HbA1c (%) | Duration of T1D (yrs) | Duration of pump therapy (yrs) | Total daily dose (U/kg /day) | Fasting C-peptide (pmol/l) |
|---|---|---|---|---|---|---|---|
| $t_{max}$ [a] | 0.06 | -0.30 | 0.45 | -0.07 | 0.07 | 0.08 | 0.16 |
| $MCR_I$ [a] | -0.35 | -0.16 | -0.45 | -0.06 | 0.16 | -0.52 | -0.19 |
| $ins_c$ [a] | 0.44 | 0.24 | 0.52 | 0.09 | 0.29 | 0.71[b] | -0.19 |

[a] Mean of the two occasions

[b] $p = 0.014$.
